# Growth Behavior of Ni on Hydrogen-Etched WS_2_ Surface

**DOI:** 10.1021/acsami.4c11506

**Published:** 2024-10-07

**Authors:** Hui-Ting Liu, Wan-Hsin Chen, Shu-Jui Chang, Chueh-Cheng Yang, Chia-Hsin Wang, Wei-Tung Liu, Kuan-Yu Chen, Naoya Kawakami, Kuan-Bo Lin, Chun-Liang Lin, Chenming Hu

**Affiliations:** † International College of Semiconductor Technology, Hsinchu 300093, Taiwan; ‡ Department of Electrophysics, National Yang Ming Chiao Tung University, Hsinchu 300093, Taiwan; § CREDM, Taiwan Semiconductor Manufacturing Company, Hsinchu 30075, Taiwan; ∥ National Synchrotron Radiation Research Center, Hsinchu 300092, Taiwan; ⊥ Department of Electrical Engineering and Computer Sciences, University of California, Berkeley, California 94720, United States

**Keywords:** TMD material, STM, XPS, metal growth behavior, sulfur vacancy, desulfurization

## Abstract

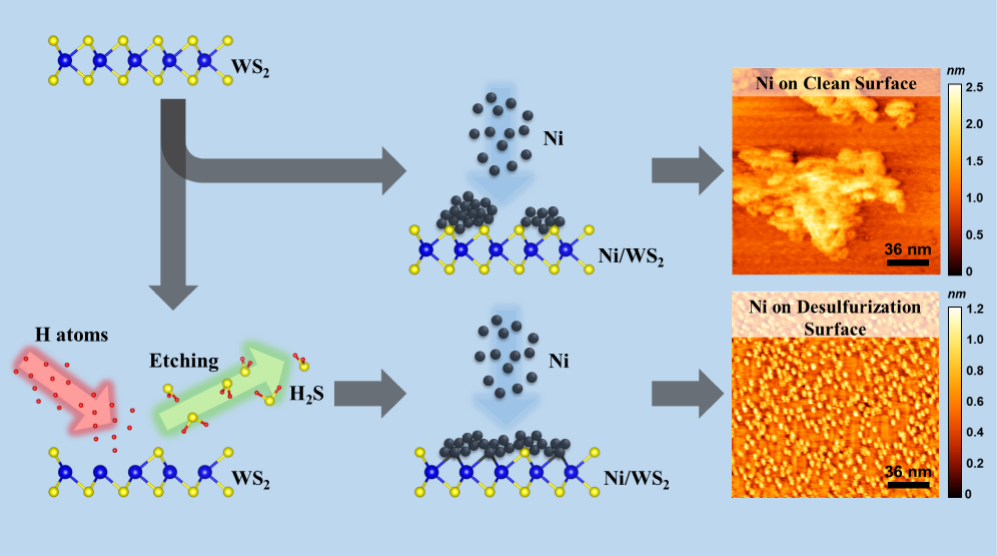

Transition metal dichalcogenides (TMDs) are 2D materials in which the layers are stacked together by van der Waals forces. Although TMDs are expected to be promising for electronic applications, forming a uniform electrode on them is challenging because of the low adhesion forces between metals and TMDs. This study focuses on improving the quality of metal electrodes by introducing atomic H to create surface defects, using Ni on WS_2_ as an example. The detailed effects of H etching and subsequent Ni growth were investigated using scanning tunneling microscopy (STM) and synchrotron-based X-ray photoemission (XPS) techniques. Our studies reveal that introducing point defects of ∼3.05 × 10^11^ cm^–2^ on the WS_2_ surface, results in a significant shift in Ni growth from the Volmer–Weber to a near Frank-van der Merwe mode. The origin of the change is the bond formation between the Ni and W atoms, which is expected to realize ohmic contact. The optimization of metal–TMD interfaces offers valuable insights for advanced applications.

## Introduction

Two-dimensional (2D) semiconductors are of great interest due to their atomic thickness and flatness, making them a promising material to promote the scaling down of modern electronic components. Among various 2D semiconductors, transition metal dichalcogenides (TMDs) provide vast possibilities to overcome the current thickness limitation in Si-based devices.^1−5^ As one of the promising functional materials of the TMD family, tungsten disulfide (WS_2_) exhibits many unique properties, such as thickness-dependent band gap,^6−9^ high carrier mobilities,^10^ large exciton binding energy,^11^ large spin–orbit splitting,^12^ and high ON/OFF current ratio.^13^ While monolayer WS_2_ holds great potential for future electronic applications, its interface with metal hinders efficient electrical transport. There are various methods to enhance the electrical transport properties, such as lowering the Schottky barrier height through doping method,^14^ improving the metal–TMD interface by inserting the Fermi-level depinning layer,^15^ and suppressing metal-induced gap states (MIGS) by introducing semimetals between the metal–TMD junction.^16,17^ A recent theoretical study based on density functional theory (DFT) predicted that sulfur vacancies (SVs) at the metal–TMD junction could reduce contact resistance and achieve ohmic contact through enhanced interlayer charge transfer.^18^ In addition, among many intrinsic defects in TMD materials, only the topmost chalcogen vacancies effectively support the metal–TMD interface contact.^18^ On the contrary, it is known that conventionally growing epitaxial metal thin films on TMD surfaces is neither easy nor efficient, especially for Ni, a widely used metal in the semiconductor industry. In most cases, the growth behavior of Ni atoms on TMD follows the Volmer–Weber (VW) mode, also known as the island growth mode, which occurs when the interfacial energy is weak and the deposited atoms are more strongly bonded to each other than to the substrate, resulting in nonuniform clusters.^19−21^ Due to the low surface energy of most TMD surfaces, growing uniform metal thin films on these materials presents a well-known challenge. Introducing surface SVs can enhance the surface energy, facilitating the growth of uniform metal thin films on TMDs. Therefore, by developing a method to create SVs on TMD surfaces without destroying their structur, it is possible to achieve both ohmic contact and uniform electrodes.

While the H_2_ anneal or H_2_ plasma process effectively removes sulfur atoms, these methods may damage the surface structure of 2D materials.^22,23^ Therefore, developing an efficient and gentle desulfurization technique that preserves the quality of the 2D materials is imperative. Atomic hydrogen (H), on the other hand, is an effective alternative. It has been confirmed that the atomic H selectively and delicately etches chalcogenide to reconstruct the surface structure of 2D materials.^24,25^

In this study, atomic H was used to create SVs on the surface of WS_2_ before Ni was deposited on top. Our *in situ* scanning tunneling microscopy (STM) observations showed that the Ni growth behavior shifted from 3D to 2D. Furthermore, *in situ* X-ray photoelectron spectroscopy (XPS) confirmed the effective formation of the covalent Ni–S and metallic Ni–W bonds at the Ni-WS_2_ interface. These findings suggest that the contact interface between the metals and WS_2_ can be optimized by creating the required number of SVs.

## Experimental Section

### STM Measurements

The high-quality 2*H*-WS_2_ crystal was purchased from 2D Semiconductors (Arizona, U.S.A.). We prepared the WS_2_ sample for STM measurement by (i) attaching WS_2_ onto the STM sample plate with silver adhesive paste. The silver adhesive was dried for 1 h by heating at 80 °C. (ii) The sample was kept in a bell jar and evacuated overnight. This step is essential to prevent significant outgassing of the silver adhesive within the ultrahigh vacuum environment of the STM chamber. (iii) The sample was loaded into the STM chamber after attaching a carbon conductive tape onto the sample surface. (iv) Before commencing measurements, we mechanically exfoliated the sample by carefully peeling off the carbon conductive tape inside the STM chamber. These steps ensure that the WS_2_ sample is firmly attached to the STM sample plate, the vacuum in the chamber is maintained, and the sample surface is free from contaminants for accurate STM measurements.

The base pressure of the STM chamber was kept below 5 × 10^–10^ Torr. A flux of atomic H was produced by dissociating H_2_ gas molecules with a hot tungsten filament in the STM chamber.^26,27^ During the desulfurization, the pressure was kept at 2 × 10^–6^ Torr. STM images were acquired at room temperature in constant-current mode using a tungsten tip.

### Synchrotron-Based XPS Measurements

The XPS experiments were conducted at Taiwan Light Source (TLS) beamline 24A1 at the National Synchrotron Radiation Research Center (NSRRC). The photon energies were fixed at 1150 and 1000 eV. The XPS spectra for each element were recorded with an energy step of 0.05 eV. The photon energies were calibrated using the Au 4*f* peak from Au foil for each sample, ensuring the precise observation of subtle variations across the samples. All spectra were collected at room temperature and under a pressure of 5 × 10^–9^ Torr.

To further investigate the interactions occurring at the Ni–WS_2_ interface, we prepared samples by depositing Ni onto the desulfurized WS_2_ surface in the STM chamber. The Ni source, which was obtained from Goodfellow Cambridge Ltd., was evaporated using a UHV Evaporator EFM3 by Scienta Omicron. We maintained a precise pressure of 1 × 10^–9^ Torr during Ni deposition, and the sample was held at room temperature throughout the process. The sample consisted of a Ni-WS_2_/sapphire structure, where the Ni layer was more than 30 nm thick, which was greater than the typical measurement depth of XPS. To observe the elements and chemical bonds at the Ni–WS_2_ interface in XPS measurements, we used silver epoxy EPO-TEK H21D to transfer the Ni–WS_2_ structure onto P^2+^ Si. After baking at 100 °C for 4 h, the structure transformed into a reversed WS_2_/Ni/P^2+^ Si once the sapphire was removed. This method proved highly effective in achieving our desired outcome.

### Simulation Method

Nanodcal was utilized to calculate the quantum transport and electrical properties of the WS_2_ transistor model.^28^ Nanodcal is a first-principles calculation package based on the DFT and NEGF-DFT formalism. Norm-conserved pseudopotentials and an LCAO basis set using the LDA-DZP function within the LDA_PZ81 functional were employed. The Monkhorst–Pack k-points mesh was set to *k* = 1 × 12 × 100. Convergence criteria for the Hamiltonian and density matrices were established at *dE* < 10^–5^ eV. The source-to-drain current points (*I*_DS_) were integrated using Landauer’s formula
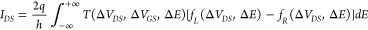
1

Where *f*_*L*_–*f*_*R*_ represents the difference in Fermi–Dirac occupation functions between the left and right Ni(111)/TMD leads. *V*_*DS*_ denotes the source-to-drain voltage, and *V*_*GS*_ represents the source-to-gate voltage. *T*(*ΔV*_*DS*_,*ΔV*_*GS*_,*ΔE*) refers to the bias-dependent transmission spectrum.

## Results

**Figure 1:**
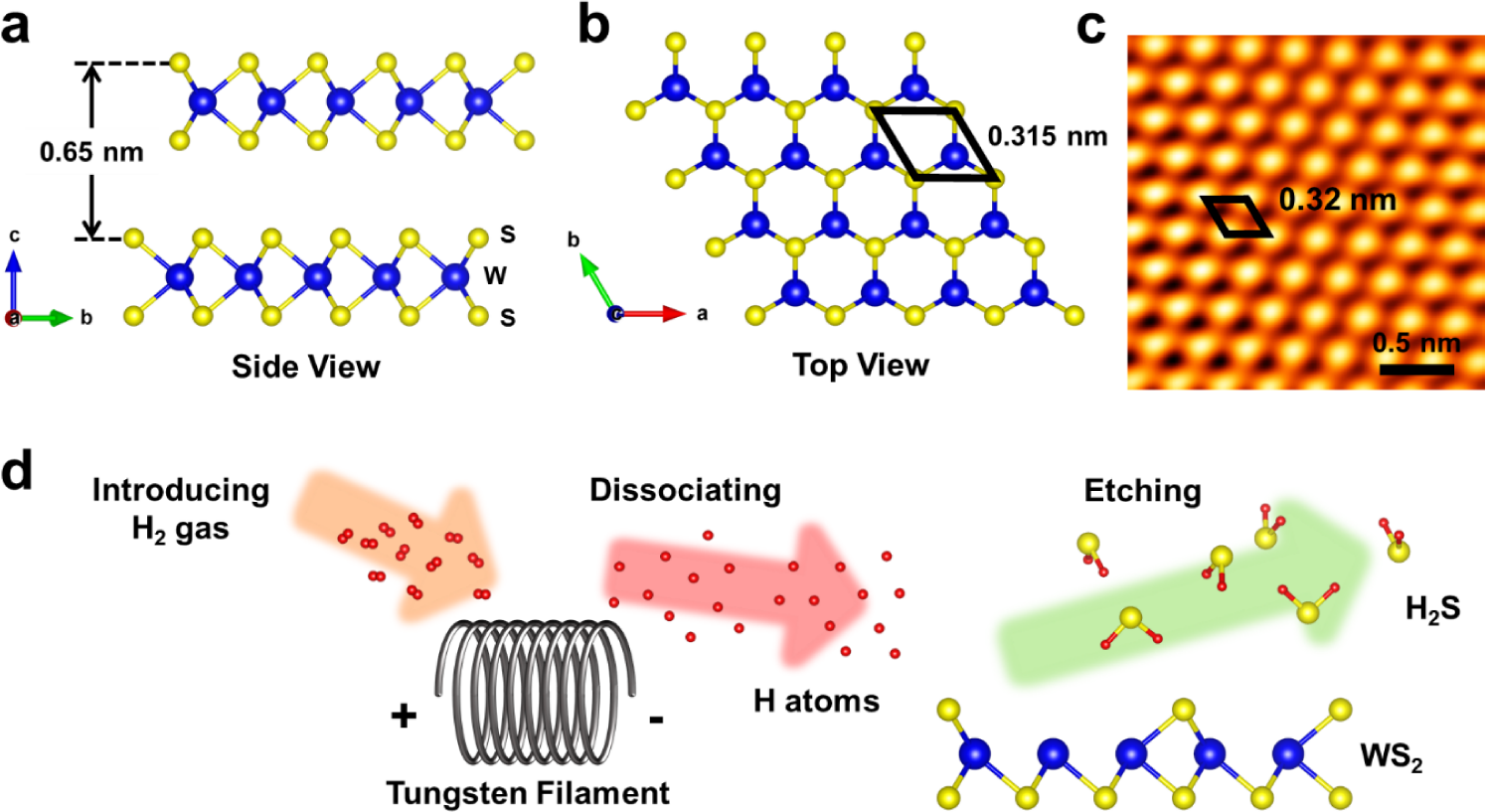
Ball model of the crystal structure, STM images of 2H-WS_2_, and the sketch of the desulfurization process. (a) The side and (b) the top view of the 2H-WS_2_ crystal. The thickness of a single layer is 0.65 nm, and the lattice constant is 0.315 nm. The W and S atoms are depicted as blue and yellow spheres, respectively. (c) Atomic-resolution STM image of as-cleaved WS_2_ (sample bias: −0.3 V, tunneling current: 0.7 nA). The protrusions represent S atoms on the surface. A black rhombus indicates the unit cell. (d) Schematic illustration of the desulfurization process.

Figure 1a,b represents a ball model of the 2H-WS_2_ from the side and top view, respectively. A layer consists of S–W–S units, which are vertically stacked by van der Waals (vdW) interaction with a spacing of 0.65 nm to form a bulk.^29,30^ The in-plane lattice constant is 0.315 nm. Figure 1c shows an atomic-resolution STM image of the pristine WS_2_ surface. The protrusions in the STM image represent S atoms located on the topmost layer of WS_2_, forming a hexagonal lattice with a lattice constant of 0.32 ± 0.05 nm.

**Figure 2:**
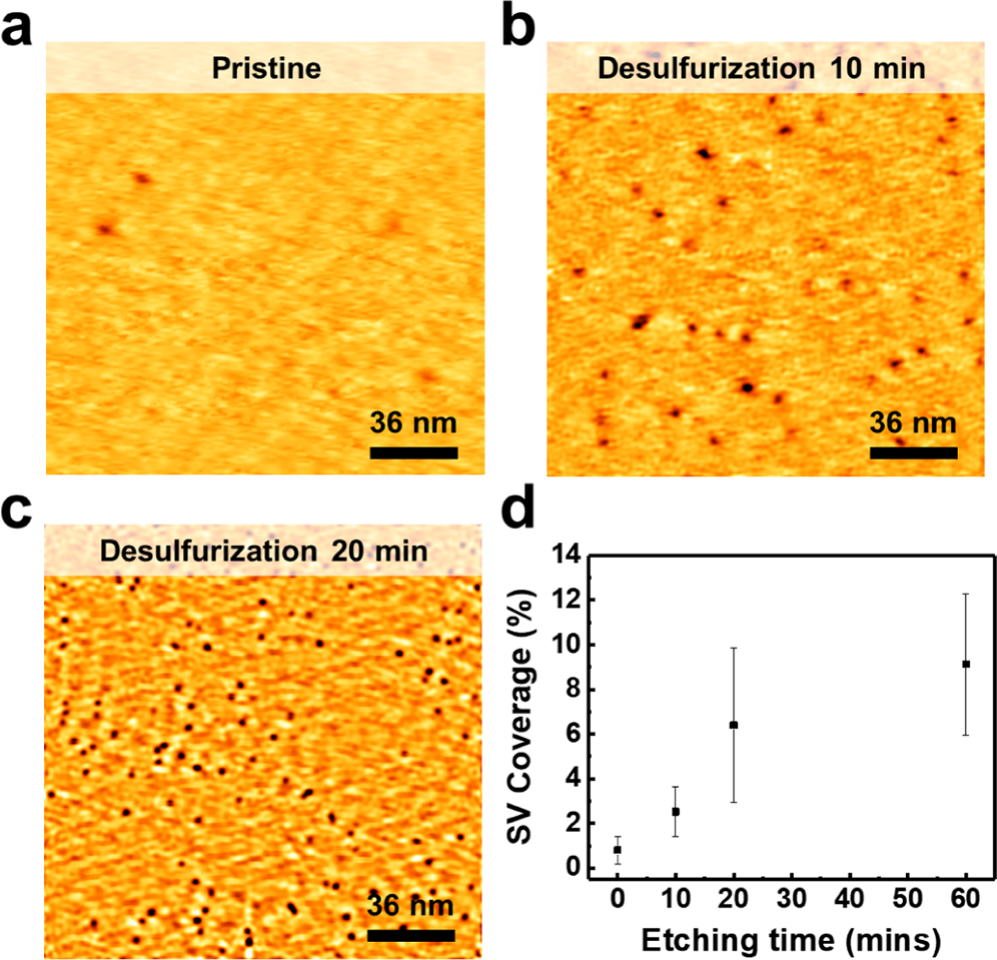
Evolution of the WS_2_ surface during the desulfurization process. The STM images of the WS_2_ surface (a) without desulfurization (sample bias: −0.5 V, tunneling current: 1.4 nA) and with desulfurization for (b) 10 (sample bias: 0.6 V, tunneling current: 0.8 nA) and (c) 20 (sample bias: 0.5 V, tunneling current: 0.5 nA) minutes. (d) The evolution of SV coverage on the WS_2_ surface.

The subsequent STM characterizations focus on exploring the growth behavior mechanism via the *in situ* desulfurization process. Figure 1d illustrates the *in situ* desulfurization process in the STM chamber. The W filament was heated above 1000 °C, causing the dissociation of molecular hydrogen to atomic hydrogen. The filament is positioned away from the sample plate to minimize the effect of heat transfer. As dissociated atomic hydrogen reaches the sample, it reacts with surface S to form hydrogen sulfide (H_2_S_(g)_). This reaction leads to the formation of SVs on the WS_2_ surface. The evolution of the sample surface by desulfurization was monitored by STM, as shown in Figure 2. The *as-cleaved* surface, shown in Figure 2a, is relatively flat and clean with few depressions representing the SVs. The SV density is about 6.94 × 10^10^ cm^–2^, corresponding to 0.8 ± 0.6% coverage. The ratio of S atoms to W atoms (S/W) in pristine WS_2_ is about 1.992, nearly approaching the perfect WS_2_ crystal with an S/W ratio = 2. Figure 2b,c shows the STM images for WS_2_ after 10 and 20 min of desulfurization. As the desulfurization proceeds, the coverage of SVs increased to 2.5 ± 1.1 and 6.4 ± 3.5%. In all the images, the SVs with similar sizes were distributed randomly on the surface. The corresponding defect density, derived from the STM images in Figure 2a–c is about 6.94 × 10^10^, 3.05 × 10^11^, and 7.92 × 10^11^ cm^–2^, respectively. Note that upon further desulfurization for 60 min, the surface with a half of chance can be destroyed as shown in Figure S1.

**Figure 3:**
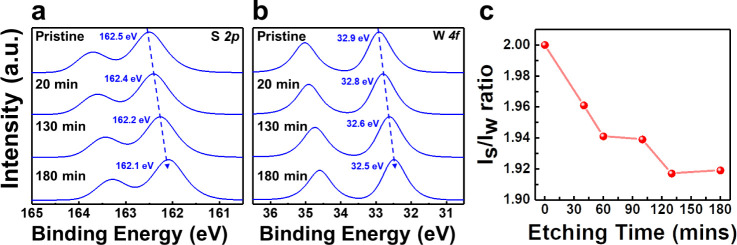
XPS spectra during the desulfurization process. XPS spectra of (a) S *2p* and (b) W *4f* core level for various desulfurization times of the WS_2_ surface. (c) The evolution of the S/W ratio with the desulfurization time, which was calculated by integrating corresponding peaks in the XPS spectra.

We further characterized the desulfurized sample by XPS, which provided the changes in the chemical environment of atoms over the entire sample. Synchrotron-based XPS was employed to analyze the number of S and W elements after the desulfurization. Figure 3a,b shows the XPS spectra of S *2p* and W *4f* spectra for the WS_2_ surface during the desulfurization for 0, 20, 130, and 180 min, respectively. The longer desulfurization shifts the binding energy of S 2*p* from 162.5 to 162.1 eV and that of W 4*f* from 32.9 to 32.5 eV. The lowering of the binding energies indicates the weakening of W–S bonds or S deficiency in WS_2_. The stoichiometric ratio of W and S is calculated by
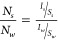
2

Where *N*_*S*(*W*)_, *I*_*S*(*W*)_, and *S*_*S*(*W*)_ represents the density, area in the spectra, and sensitivity factor of S (W) atoms, respectively. The sensitivity factor  is determined by asssuming that the  of pristine WS_2_ is equal to 2. Figure 3c shows the evolution of the ratio between S and W with the desulfurization time. The proportion of S atoms relative to W atoms decreases, indicating the formation of SVs. Two saturated plateaus can be found in Figure 3c, offering insights beyond STM observations. While STM can visualize the distribution of SVs on the surface, it is difficult to quantitatively determine the desulfurization especially after the TMD structure is destroyed. Here, by using XPS, we can overcome this limit and provide more information. The first plateau in Figure 3c can be explained by the gradual saturation of SVs on the WS_2_ surface within the TMD structure. The second plateau indicates the saturation of desulfurization following the destruction of the TMD structure of WS_2_. It is important to note that the difference in the desulfurization timeline between the XPS and STM experiments is due to the use of different equipment. Both STM and XPS results confirmed that desulfurization effectively removed S atoms from the WS_2_ surface.

**Figure 4:**
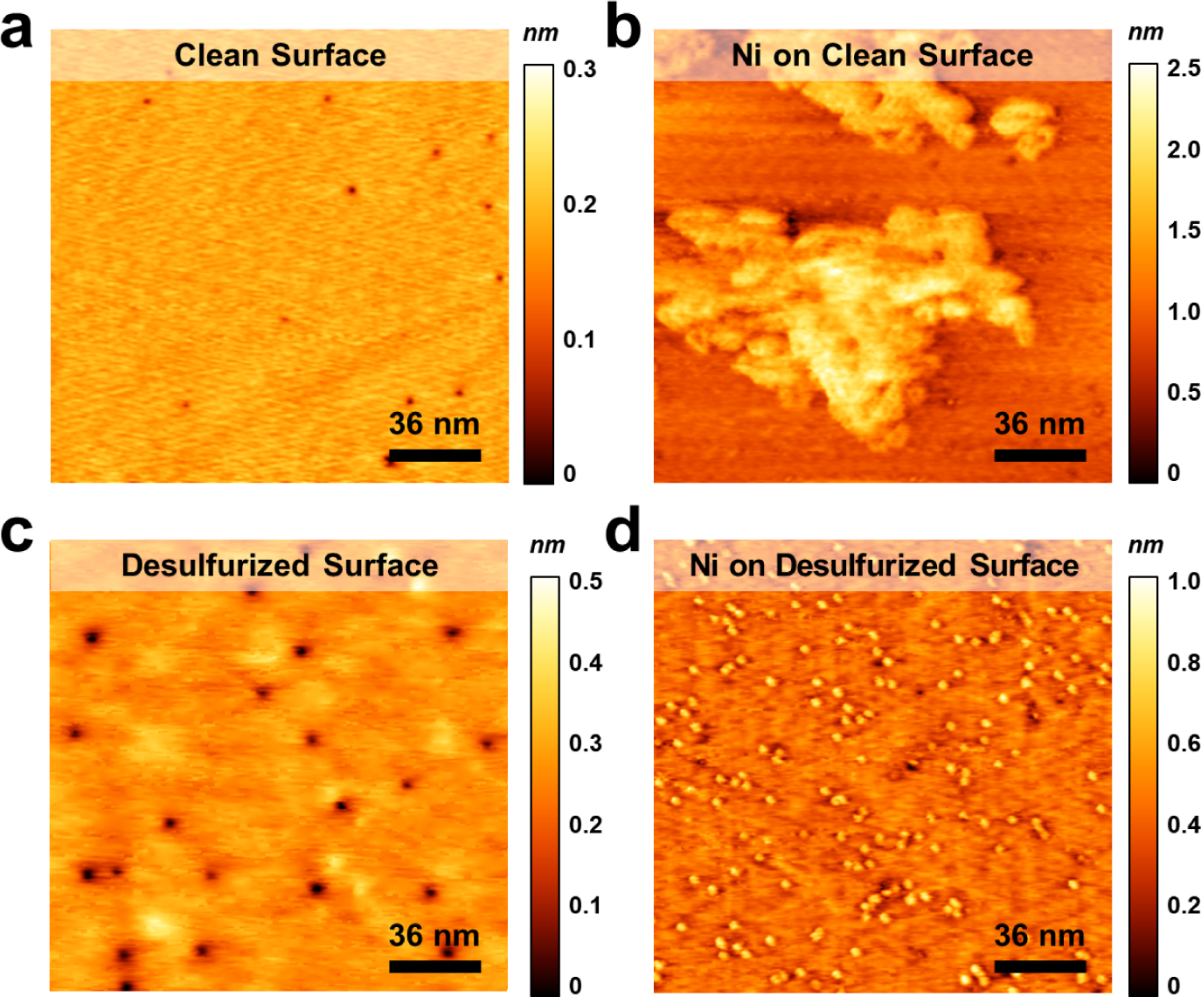
STM images of Ni on WS_2_ with and without desulfurization. STM images of (a) pristine WS_2_ surface (sample bias: −1.0 V, tunneling current: 1.0 nA), (b) Ni on pristine WS_2_ surface (sample bias: −0.9 V, tunneling current: 1.0 nA), (c) desulfurized WS_2_ surface (sample bias: −1.0 V, tunneling current: 1.3 nA), and (d) Ni on desulfurized WS_2_ surface (sample bias: −0.5 V, tunneling current: 0.3 nA).

We further investigated how the desulfurized WS_2_ surface affects the growth mechanism of Ni, providing insights into the natural properties of growing metal electrode in 2D systems.^31,32^ Before Ni deposition, the surface of WS_2_ was flat and clean, as shown in Figure 4a. Subsequently, Ni atoms were deposited on the pristine WS_2_ surface for 30 min. The resulting STM image is shown in Figure 4b. The Ni clusters are irregular in shape with a lateral dimension of around 150 nm and a height of approximately 2.5 nm. These clusters were randomly distributed on the WS_2_ surface. The 3D growth of Ni clusters suggested a weak interaction between Ni and the pristine WS_2_ surface. The 3D growth of Ni clusters suggested a weak interaction between Ni and the pristine WS_2_ surface, which may lead to suboptimal contact between the metals and 2D materials.

After 20 min of desulfurization, the WS_2_ surface exhibits more SVs than the pristine surface, as shown in Figure 4c. Following a 30-min Ni deposition on the desulfurized WS_2_ surface, Ni clusters with a diameter of approximately 3 nm and a height of 1 nm were uniformly distributed, as shown in Figure 4d. It indicates that the growth behavior of Ni on the desulfurized WS_2_ surface was significantly different. The significant modification of the growth behavior due to desulfurization is expected to enhance the contact between the metals and 2D materials.

**Figure 5:**
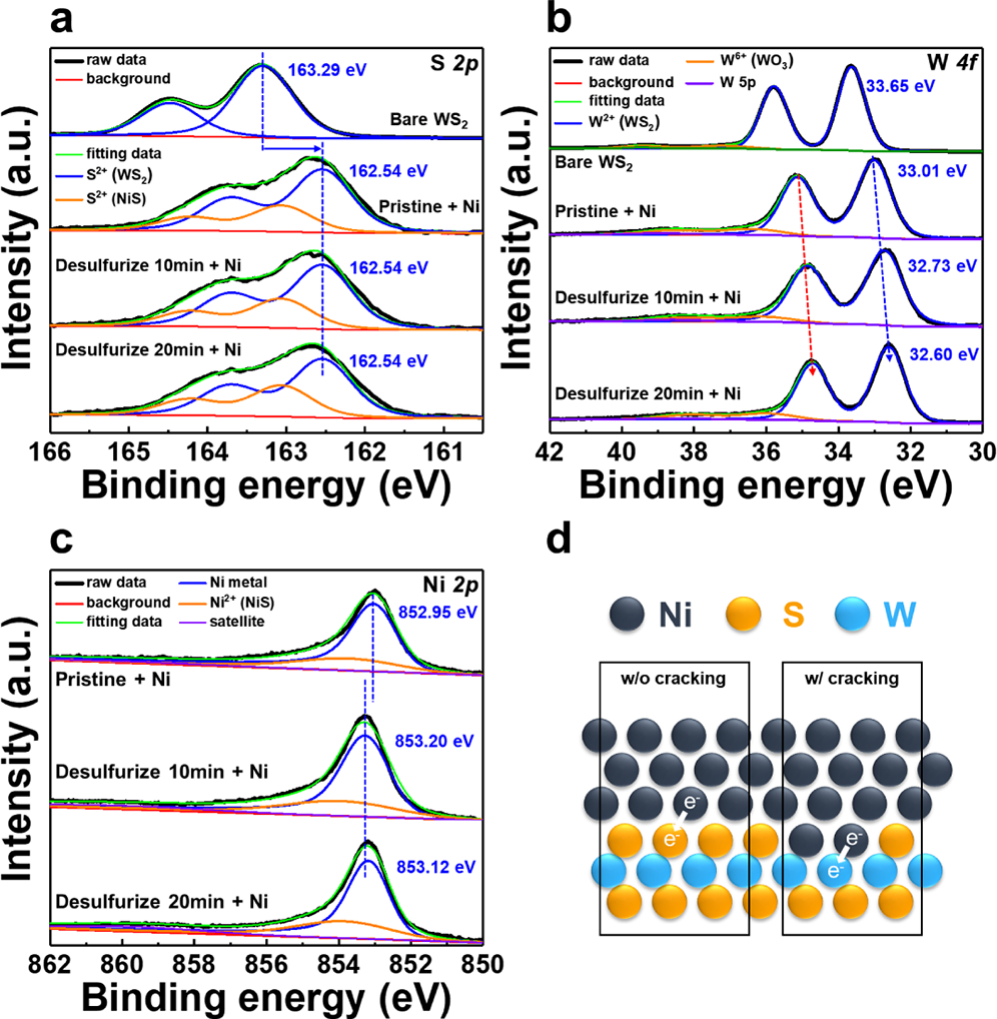
XPS spectra of Ni on desulfurized WS_2_. XPS spectra of (a) S 2*p*, (b) W 4*f*, and (c) Ni 2*p* core level for various desulfurization times, as indicated in the figures. (d) Schematic illustration showing the charge transfer between Ni–S and Ni–W.

The details of the chemical interactions at the interface between Ni and WS_2_ were investigated using XPS. Figure 5 shows the XPS spectra for the Ni/WS_2_ structure, where the WS_2_ was subjected to various desulfurization times of 0, 10, and 20 min. The photon energies for the W *4f* and S *2p* spectra were fixed at 1150 eV, while that of Ni *2p* was fixed at 1000 eV. The photon energies were adjusted to eliminate interference from deeper Ni signals in Ni/WS_2_, enhancing the interface signal. The XPS spectra for Ni *2p* using 1150 eV are shown in Figure S2. Figure 5a-c shows the XPS spectra of S *2p*, W *4f*, and Ni *2p* for the Ni/WS_2_ structure. The curves were fitted using fixed full width at half maximum values for W and S. Ni deposition on pristine WS_2_ causes both the S *2p* and W *4f* signals to shift toward lower binding energies, which can be attributed to the hybridization between the metal and the semiconductor.^33^ Moreover, the appearance of additional peaks shown by the orange curves indicates the formation of chemical bonds between the Ni and S atoms.

The desulfurization process does not affect the S 2*p* signal, while the binding energy of W 4*f* and Ni 2*p* shifts toward the lower and higher values, respectively. It infers the charge transfer from Ni to W on a desulfurized surface. Figure 5d illustrates the transformation of the Ni/WS_2_ interface via desulfurization. As the desulfurization time becomes longer, the intensity of the new peak gets stronger, indicating that more S atoms are in contact with the Ni atoms. It is consistent with the STM measurements, which indicate that the Ni atoms exhibit 2D growth behavior. At the same time, the Ni atoms interact with W atoms to form metallic bonds, with electron transfer occurring from Ni to W because of the larger electronegativity of W (2.36) compared to that of Ni (1.92). To further investigate how these metallic bonds influence the contact properties, we perform first-principles calculation based on density functional theory (DFT) and a nonequilibrium Green’s function (NEGF) method to evaluate the *I–V* characteristics of the WS_2_ transistor with Ni–S and Ni–W contacts. As demonstrated in Figure S3, the *I–V* behavior of the Ni–W contact property is closer to the ideal ohmic behavior than that of the Ni–S contact.

**Figure 6:**
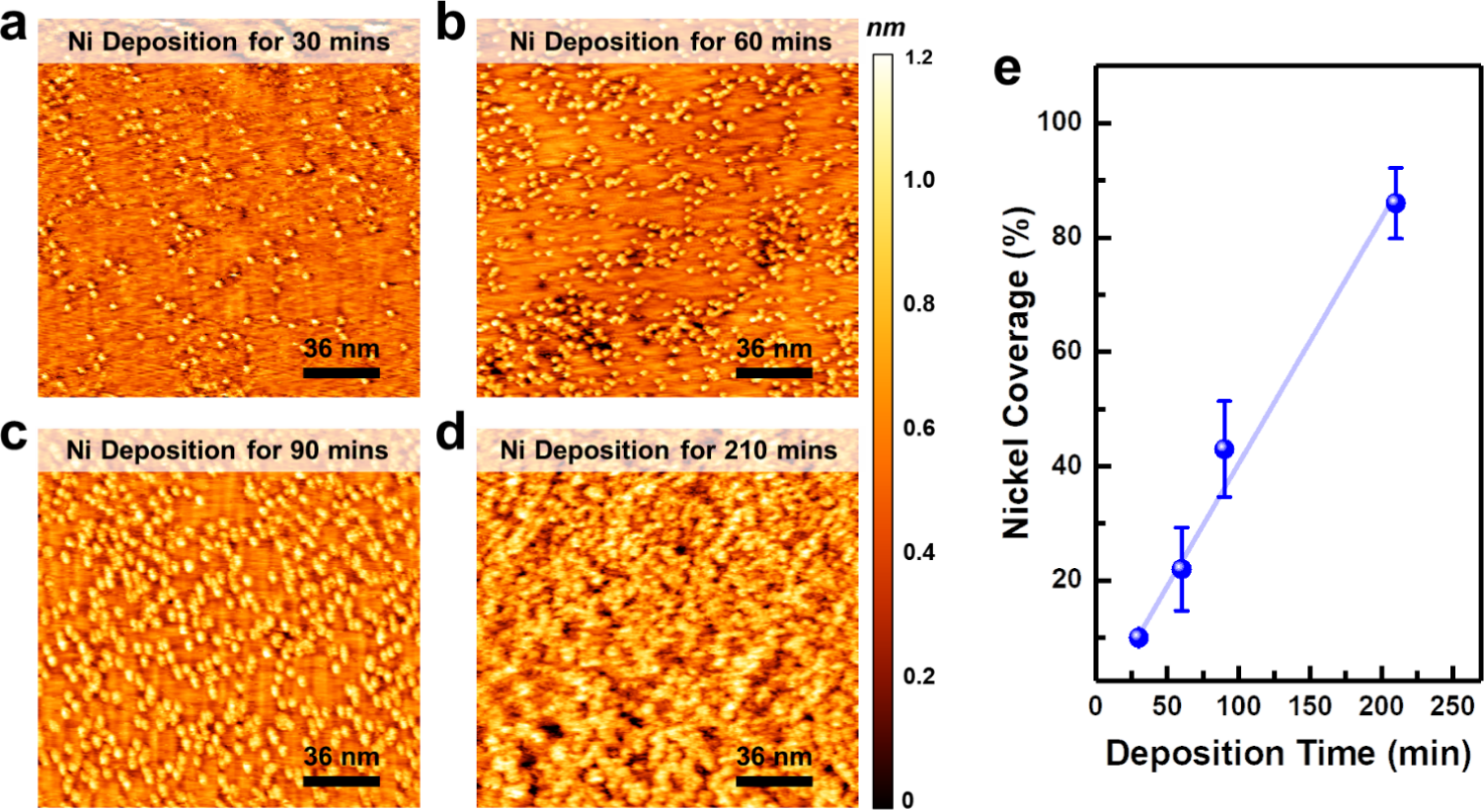
The evolution of STM images by the Ni deposition on the desulfurized WS_2_ surface. STM images of Ni on a desulfurized WS_2_ surface for the deposition times of (a) 30 (sample bias: −0.5 V, tunneling current: 0.3 nA), (b) 60 (sample bias: −0.5 V, tunneling current: 0.3 nA), (c) 90 (sample bias: −0.5 V, tunneling current: 0.3 nA), and (d) 210 (sample bias: −0.5 V, tunneling current: 0.3 nA) minutes. (d) The evolution of Ni coverage with deposition time on the desulfurized WS_2_ surface.

We observed the detailed evolution of Ni clusters on a surface desulfurized for 20 min using STM, as shown in Figure 6a–d, for the growth times of 30, 60, 90, and 210 min. As seen in the STM images in Figure 6a, during the initial 30 min, Ni clusters formed randomly on the WS_2_ surface, with a height of approximately 1 nm (equivalent to three layers of Ni). The positions of Ni clusters reflect those of SVs, as the apparent number of SVs is reduced from the desulfurized surface. After 60 min of deposition, as shown in Figure 6b, more Ni clustersformed, though their size remained nearly identical to those observed at 30 min. When the deposition time increased to 90 min, the Ni clusters began to evolve laterally while maintaining a height of approximately 1 nm, as shown in Figure 6c. After 210 min of deposition, the Ni islands merged to form a uniform film that covered 86 ± 6.2% of the surface, as shown in Figure 6d. The coverage change is summarized in Figure 6e. This growth pattern is similar to the Frank–van der Merwe (FM) growth mode, also known as the layer-by-layer growth, which occurs when the interfacial energy between the substrate and the upper layer material is predominant. In our case, this is attributed to the sulfur vacancies created by the hydrogen etching process, which enhances the Ni-WS_2_ bonding.

Through the STM and XPS observations, we revealed that defects on the surface of 2D materials significantly improve their contact with metals. The change in the growth mode has also been noticed on the HOPG surface.^34^ Therefore, this method can be used for a wide range of 2D materials. Moreover, atomic hydrogen is a promising approach for TMDs, as it reacts with various chalcogen species.^35^ The alteration in growth behavior may be correlated to the formation of metallic bonds between Ni and W, as evidenced in Figure 5. The Ni atoms forming a metallic bond with the W atoms at the SVs serve as a seed for the lateral growth of Ni clusters, achieving the FM growth mode.

## Conclusion

In this work, we demonstrated a method to control the growth behavior of metal on TMDs using Ni and WS_2_ as an example. The growth behavior dramatically changes from the VW mode to the FM mode after the introduction of SVs on the WS_2_ surface by desulfurization. By monitoring the evolution of Ni growth by STM, we clarified that SVs on the WS_2_ surface act as the center for the nucleation of Ni atoms and lead to a change in the growth behavior. In addition, the hybridization between the Ni film and W atoms may give rise to ohmic contact at the interface. Our research demonstrated a relatively simple and practical method for improving the metal–TMD contact, which also suggests promising applications for TMD materials.

## Supporting Information Available

STM image of 60 min desulfurization, illustration of the probing depth of XPS and the DFT calculation to evaluate the contact properties of the WS_2_ transistor with and without desulfurization (PDF)
