# Supplementary material for: Growth Behavior of Ni on Hydrogen-Etched WS2 Surface
Source: ACS Appl Mater Interfaces. 2024 Oct 7;16(41):56336–42. doi: 10.1021/acsami.4c11506 (PMC11492163; doi:10.1021/acsami.4c11506)
Supplement: Supplementary file 1 — am4c11506_si_001.pdf [file am4c11506_si_001.pdf]

Supporting Information :

Growth behavior of Ni on hydrogen-etched WS_2 surface

Hui-Ting Liu[1, 2], Wan-Hsin Chen[2], Shu-Jui Chang[3], Chueh-Cheng Yang[4], Chia-Hsin Wang[4], Wei-Tung Liu[2], Kuan-Yu Chen[2], Naoya Kawakami[2], Kuan-Bo Lin[1], Chun-Liang Lin[2*], Chenming Hu[1, 5]*

* Shu-Jui Chang, SJCHANGX@tsmc.com

* Chun-Liang Lin, clin@nycu.edu.tw

[1]International College of Semiconductor Technology, Hsinchu 300093, Taiwan

[2]Department of Electrophysics, National Yang Ming Chiao Tung University, Hsinchu 300093, Taiwan

[3]CREDM, Taiwan Semiconductor Manufacturing Company, Hsinchu 30075, Taiwan

[4]National Synchrotron Radiation Research Center, Hsinchu 300092, Taiwan

[5]Department of Electrical Engineering and Computer Sciences, University of California, Berkeley, California 94720, USA

STM image of WS_2 surface after 60 mins desulfurization

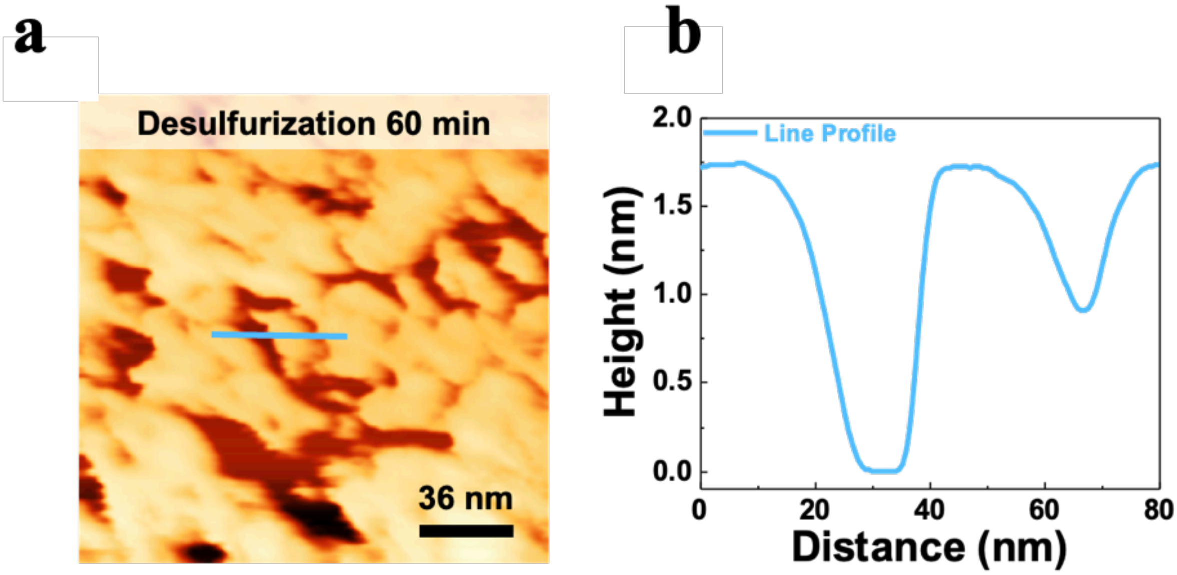

Figure S1. (a) The STM images of the WS_2 surface with desulfurization time for 60 minutes. (b) The profile at blue line in WS_2 STM result.

Figure S1 shows the WS_2 surface after 60 minutes of desulfurization. It can be observed that the WS_2 structure becomes quite irregular, with many depressed areas appear. A depth profile of one of these areas is shown in Figure S1(b). The depth is approximately 1.7 nm, which is greater than the thickness of monolayer WS_2. This result demonstrates that the WS_2 TMD structure is destroyed after 60 minutes of desulfurization.

XPS results of different photon energy

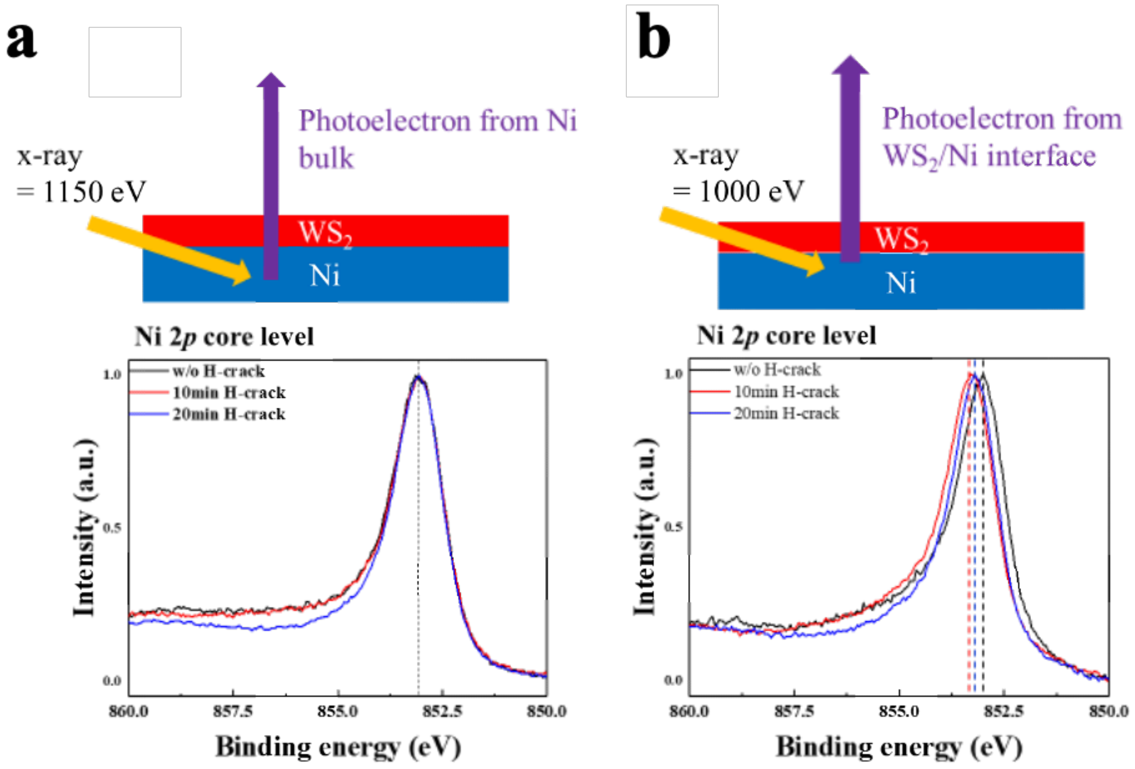

Figure S2. Illustration of the probing depth of XPS for Ni 2*p* with the photon energy at (a) 1150 eV and (b) 1000 eV.

Figure S2 shows the measurement structure of the Ni/WS_2 samples. The upper layer consisted of a monolayer WS_2, while the lower layer was the Ni metal. A correlation can be observed between the measurement depth of the sample and the incident x-ray energy. There is no observable change in the position of the Ni main peak for different desulfurization times when utilizing the x-ray at 1150 eV. However, when the x-ray energy was reduced from 1150 eV to 1000 eV, the peak position of Ni shifted for different desulfurization times. This observation confirms the existence of charge transfer behavior within the Ni-WS_2 interface.

Simulation for WS_2 transistor with different contacts

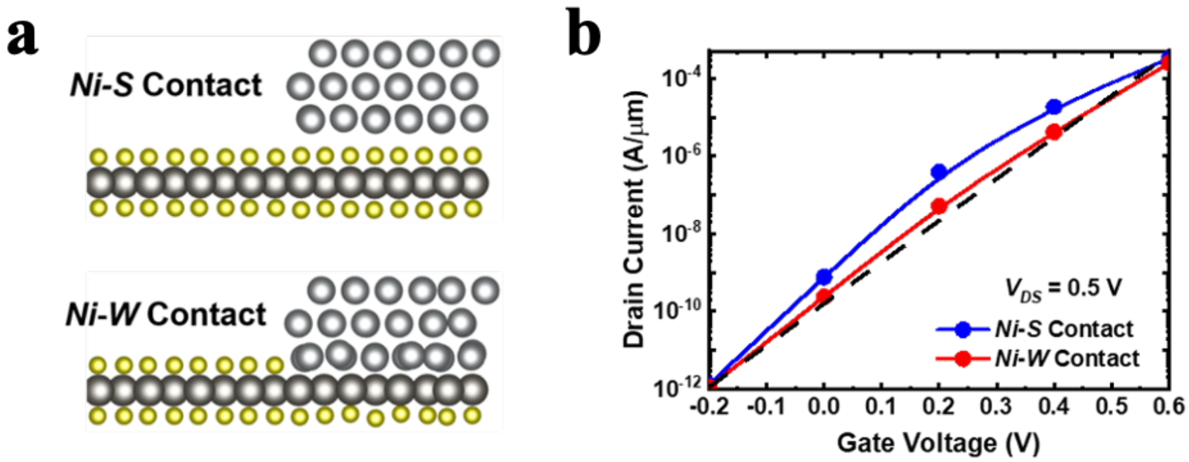

Figure S3. (a) Ni-S (up panel) and Ni-W (down panel) atomic contact models. (b) I_{DS}-V_{GS} plot of WS_2 transistor with two types of contact in (a). The black dash line is the ideal ohmic behavior.

Figure S3 shows the result of first-principles calculation based on density functional theory (DFT) and a non-equilibrium Green's function (NEGF) method to evaluate the *I*-*V* characteristics of the WS_2 transistor with Ni-S and Ni-W contacts. Figure S3(a) draws two types of the atomic contact model. Figure S3(b) showcases the I_{DS}-V_{GS} plot of the WS_2 transistor with Ni-S and Ni-W contacts, where the *I*-*V* behavior of the Ni-W contact transistor (red solid line) is closer to the ideal ohmic behavior (black dashed line) than the Ni-S contact transistor (blue solid line).
